# Morphology-Controlled Ultralow Aqueous Friction in Cationic–Anionic Mixed Surfactant Systems

**DOI:** 10.1021/acs.langmuir.6c02004

**Published:** 2026-08-24

**Authors:** Linyi Zhu, Nir Kampf, Maozhang Tian, Qun Zhang, Yaxun Fan, Yilin Wang, Jacob Klein

**Affiliations:** † Department of Molecular Chemistry and Materials Science, 34976 Weizmann Institute of Science, Rehovot 76100, Israel; ‡ Centre for Osteoarthritis Pathogenesis versus Arthritis, Kennedy Institute of Rheumatology, NDORMS, University of Oxford, Oxford OX3 7FY, U.K.; § State Key Laboratory of Enhanced Oil Recovery, 205361 Research Institute of Petroleum Exploration and Development, CNPC, Beijing 100083, P. R. China; ∥ 53030 University of Science and Technology of China, Hefei 230026, P. R. China; ⊥ State Key Laboratory of Bioinspired Interfacial Materials Science, Suzhou Institute for Advanced Research, University of Science and Technology of China, Suzhou 215123, P. R. China; # Physical and Theoretical Chemistry Laboratory, Oxford University, Oxford OX1 3QZ, U.K.

## Abstract

Aqueous boundary lubrication depends critically on stable, hydrated interfacial layers, yet the role of molecular self-assembly in the tribological performance remains insufficiently understood. Here, we investigate a catanionic surfactant system, C_12_C_3_C_12_(SO_3_)_2_/CTAB, in which controlled variation of the gemini surfactant C_12_C_3_C_12_(SO_3_)_2_ molar fraction (*X_g_* = 0.1, 0.2, 0.3) at a fixed total concentration of 2 mM drives structural transitions from spherical micelles to wormlike micelles and vesicles. Surface force balance and atomic force microscopy measurements reveal that all morphologies provide ultralow friction coefficients (10^–3^–10^–4^) via hydration lubrication but exhibit markedly different load-bearing capacities and mechanical stability. Spherical micelles provide the highest resilience and stability under pressures up to ∼70 atm; wormlike micelles display intermediate stability with partial structural damage under confinement; and vesicles yield the lowest friction (μ ≈ 10^–4^) but collapse at moderate pressures (15–30 atm). These findings demonstrate that aggregate morphology, controlled by stoichiometric charge neutralization, governs the balance between friction reduction and mechanical robustness, and highlight mixed surfactant self-assembly as an efficient strategy for low-concentration water-based lubricants.

## Graphical abstract

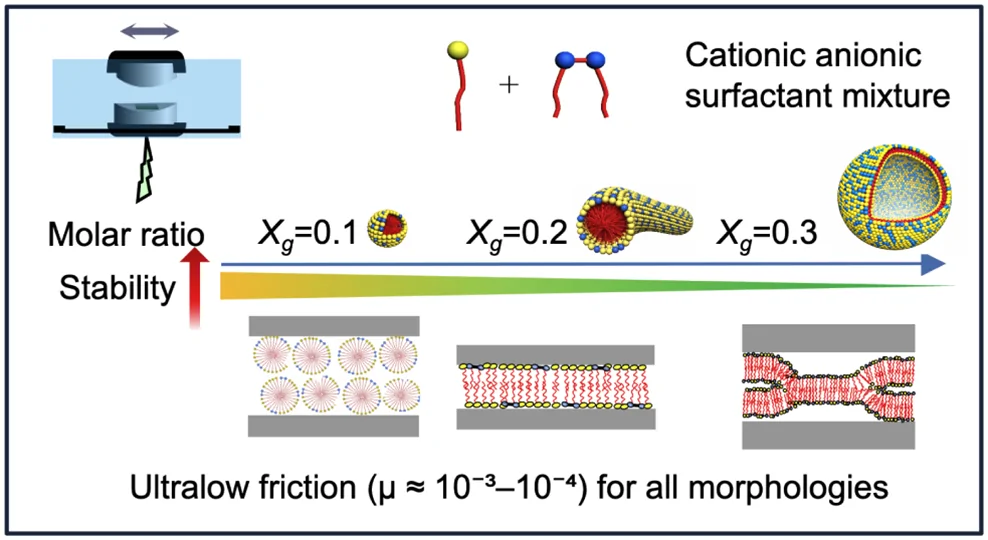

## Introduction

Aqueous lubrication is essential in natural and engineering systems, such as synovial joints, microfluidic devices, and precision manufacturing processes, where low friction must be maintained under high load and confined conditions.^1–6^ In such environments, water alone is often insufficient, so surfactants are therefore used as lubricating additives because they form highly hydrated, ordered boundary layers at interfaces.^7–10^ The unique amphiphilic nature of surfactants allows them to spontaneously self-assemble into a variety of aggregate structures in bulk solution and at interfaces.^11–13^ Such hydrated boundary layers can effectively reduce the friction coefficient.^14–16^ In surfactant-based aqueous systems, this behavior is commonly attributed to hydration lubrication,^17^ in which water strongly bound to charged headgroups forms fluid-like, mobile hydrated shells. These layers resist squeeze-out under pressure and act as load-bearing layers between sliding surfaces with minimal energy dissipation, thus dramatically reducing friction.^17^

Conventional single-chain surfactants often face challenges such as high critical micelle concentration (CMC), limited aggregate structural diversity, and sensitivity to high counterion levels,^18–20^ which collectively increase costs and restrict their efficacy in lubrication. To overcome this, mixed surfactant systems, particularly those combining cationic and anionic surfactants, have attracted considerable attention.^21–24^ Owing to synergistic electrostatic and hydrophobic interactions, such systems exhibit markedly reduced CMCs, enhanced interfacial activity, and rich self-assembly behavior, enabling the formation of complex self-assembled structures such as wormlike micelles and bilayer vesicles.^25,26^

Gemini surfactants further amplified these advantages. They comprise two hydrophobic tails and two headgroups connected through a spacer and exhibit remarkably low CMCs and strong aggregation behaviors.^27–31^ The adjustable length and rigidity of the spacer expand the range of packing parameters and enable the formation of diverse aggregate structures not readily accessible by single-chain surfactants.^30,32,33^ In particular, gemini surfactants can modify interfacial hydration, because their multivalent headgroups are often more strongly hydrated than those of conventional monomeric surfactants. For example, the anionic gemini surfactant 1,3-bis­(N-dodecyl-N-propanesulfonate sodium)-propane (C_12_C_3_C_12_(SO_3_)_2_), whose molecular structure is shown in Figure 1A, exhibits rich aggregation behavior that can be modulated by external variables such as pH, ionic strength, and counterion binding.^34,35^ When mixed with the cationic surfactant cetyltrimethylammonium bromide (CTAB), it forms a well-defined catanionic surfactant system with enhanced and tunable self-assembly behavior.^29^ Strong electrostatic attraction between oppositely charged headgroups, combined with cooperative hydrophobic interactions, enables precise control of aggregate morphology via the molar ratio, with reversible transitions among spherical micelles, wormlike micelles, and vesicles occurring without significant precipitation, even near charge neutrality.^29^

**Figure 1:**
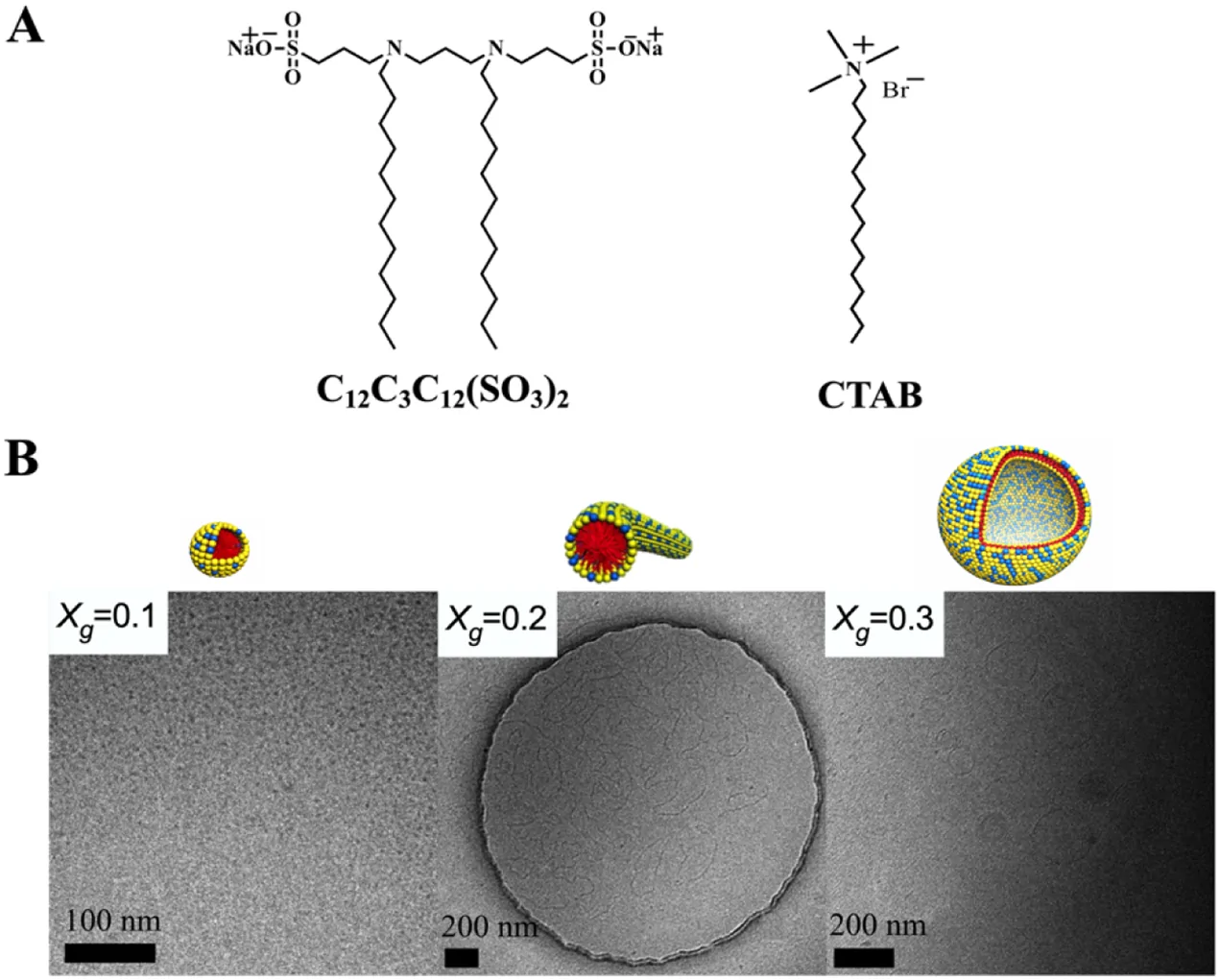
(A) Chemical structures of C_12_C_3_C_12_(SO_3_)_2_ and CTAB. (B) Representative cryo-TEM images and schematic illustrations of aggregates formed at *X_g_* = 0.1, 0.2, and 0.3, corresponding to spherical micelles, wormlike micelles, and vesicles, respectively. Cryo-TEM images are reprinted with permission from ref 29. Copyright 2013 American Chemical Society.

Despite extensive studies on the bulk aggregation of gemini-based mixed surfactant systems, the impact of distinct self-assembled structures on lubrication at solid–liquid interfaces remains poorly understood. Here, we hypothesize that combining CTAB with an anionic gemini surfactant enables synergistic adsorption and hydration, providing comparable or improved lubrication at lower total surfactant concentrations than with CTAB alone. In this study, we use the C_12_C_3_C_12_(SO_3_)_2_/CTAB mixture as a model system to investigate how bulk aggregate structure influences interfacial and tribological performance. At a total surfactant concentration of 2 mM, the system forms predominantly spherical micelles at molar fractions of C_12_C_3_C_12_(SO_3_)_2_ (*X*_*g*_) ≈ 0–0.16, wormlike micelles at *X*_*g*_ ≈ 0.16–0.22, and vesicles at *X*_*g*_ ≈ 0.22–0.43. These morphology windows were previously established using turbidity, rheology, dynamic light scattering, cryo-transmission electron microscopy (cryo-TEM), and isothermal titration calorimetry measurements.^29^ Accordingly, *X*_*g*_ = 0.1, 0.2, and 0.3 were selected as representative compositions for the three morphologies, respectively. All are below the stoichiometric neutralization point (*X*_*g*_ = 0.33) and thus retain a net positive aggregate charge. Figure 1B shows representative cryo-TEM images and corresponding schematic illustrations of the three aggregate structures.

Surface force balance (SFB) and atomic force microscopy (AFM) measurements were used to relate these distinct aggregate morphologies to their interfacial structures, frictional response, and mechanical resilience under confinement. All three compositions provide ultralow friction coefficients (10^–3^–10^–4^), but their load-bearing capacity and reversibility differ markedly. Spherical micelles demonstrated superior stability under load, whereas vesicles provided the lowest friction but suffered structural collapse under moderate pressure. These findings offer a molecular-level understanding of how self-assembled architecture can be leveraged to engineer high-performance aqueous lubricants and may offer new insights into the applications of surfactant mixtures in water-based lubrication.

## Experimental Section

### Materials and Reagents

The gemini surfactant C_12_C_3_C_12_(SO_3_)_2_ was synthesized according to a previously reported method.^34^ CTAB was purchased from TCI (Japan) with a stated purity greater than 99% and used without further purification. Both surfactants were from the same batches used in ref 29. Ultrapure water (Barnstead Nanopure Diamond) with a total organic carbon content below 1 ppb and a resistivity of 18.2 MΩ·cm was used in all experiments. All experiments were conducted at 25 ± 0.5 °C. Mica (ruby muscovite, grade I) was obtained from S&J Trading (New York, NY) and cleaved to yield atomically smooth surfaces on both sides. EPON 1004 (Shell Chemicals) glue was used to attach the mica sheets to the cylindrical lenses used in the SFB experiments.

### Preparation of Surfactant Mixture Solutions

Stock solutions of C_12_C_3_C_12_(SO_3_)_2_ and CTAB were prepared separately. Mixed surfactant solutions were obtained by mixing the stock solutions at the desired molar fractions (*X*_*g*_ = 0.1, 0.2, and 0.3) at pH 9.5, where the sulfonate groups of C_12_C_3_C_12_(SO_3_)_2_ are fully deprotonated and each molecule carries two negative charges. The mixture was then diluted to a total surfactant concentration of 2 mM, filtered to remove debris or any undissolved material, and equilibrated at room temperature for at least 24 h before measurements to ensure complete self-assembly. Small aliquots collected before and after filtration were used to measure aggregate size to confirm the formation of the expected assemblies at each molar ratio.

### Dynamic Light Scattering (DLS)

The hydrodynamic sizes of aggregates in mixed solutions were measured using a Malvern Zetasizer Nano ZS. A 633 nm He–Ne laser was used as the light source. Each measurement was performed at least five times to ensure reproducibility. The temperature was maintained at 25.0 ± 0.1 °C throughout the measurements.

### Atomic Force Microscopy Imaging of Adsorbed Structures on Mica Surfaces

AFM experiments were performed using an MFP-3D SA microscope (Asylum Research, Santa Barbara, CA) in tapping mode. Surfactant solutions were added to freshly cleaved mica substrates, and imaging was conducted within the surfactant dispersions. A Bruker SNL probe, equipped with a silicon tip (radius: 2 nm) mounted on a triangular silicon nitride cantilever (spring constant: 0.35 N/m, frequency ∼ 70 kHz), was used to scan the samples.

### Surface Force Balance Measurements

The SFB technique and the detailed procedures for measuring normal (*F*_*n*_) and shear (*F*_*s*_) forces have been described previously.^36^ A schematic of the SFB setup is shown in Figure 2. Briefly, molecularly smooth, back-silvered mica sheets (thickness ∼ 2–3.5 μm) were glued onto plano-cylindrical fused silica lenses. The mica-coated lenses were mounted in the SFB in a crossed-cylinder configuration. The separation distance (*D*) between the mica surfaces and the mean radius of curvature (*R)* were determined from the optical interference fringes of equal chromatic order (FECO) generated when white light was transmitted through the back-silvered mica sheets. The normal force was calculated as *F*_*n*_ *= K*_*n*_Δ*D*, where Δ*D* is the bending of the normal spring and *K*_*n*_ is the spring constant (127 N/m). The upper lens was attached to a four-sectored piezoelectric tube (PZT), providing highly parallel, precisely controlled lateral motion (*x*_0_). The shear force *F*_*s*_ *= K*_*s*_Δ*x* was determined from the bending (Δ*x*) of orthogonal springs (*K*_*s*_ = 375 N/m) using an air-gap capacitor probe. Normal and shear interactions between two bare mica surfaces were first measured and calibrated in air and pure water as controls. The measurement in pure water provided the reference bare mica-water double-layer interaction and established the zero-separation distance before introducing the surfactant dispersion.^15^ The water was then removed via a pipe connected to the SFB chamber, and the mica surfaces were incubated overnight in 2 mM surfactant dispersions (*X*_*g*_ = 0.1, 0.2, and 0.3) before measurements. Measurements at each ratio were repeated in at least two independent experiments, with three different contact regions analyzed per experiment.

**Figure 2:**
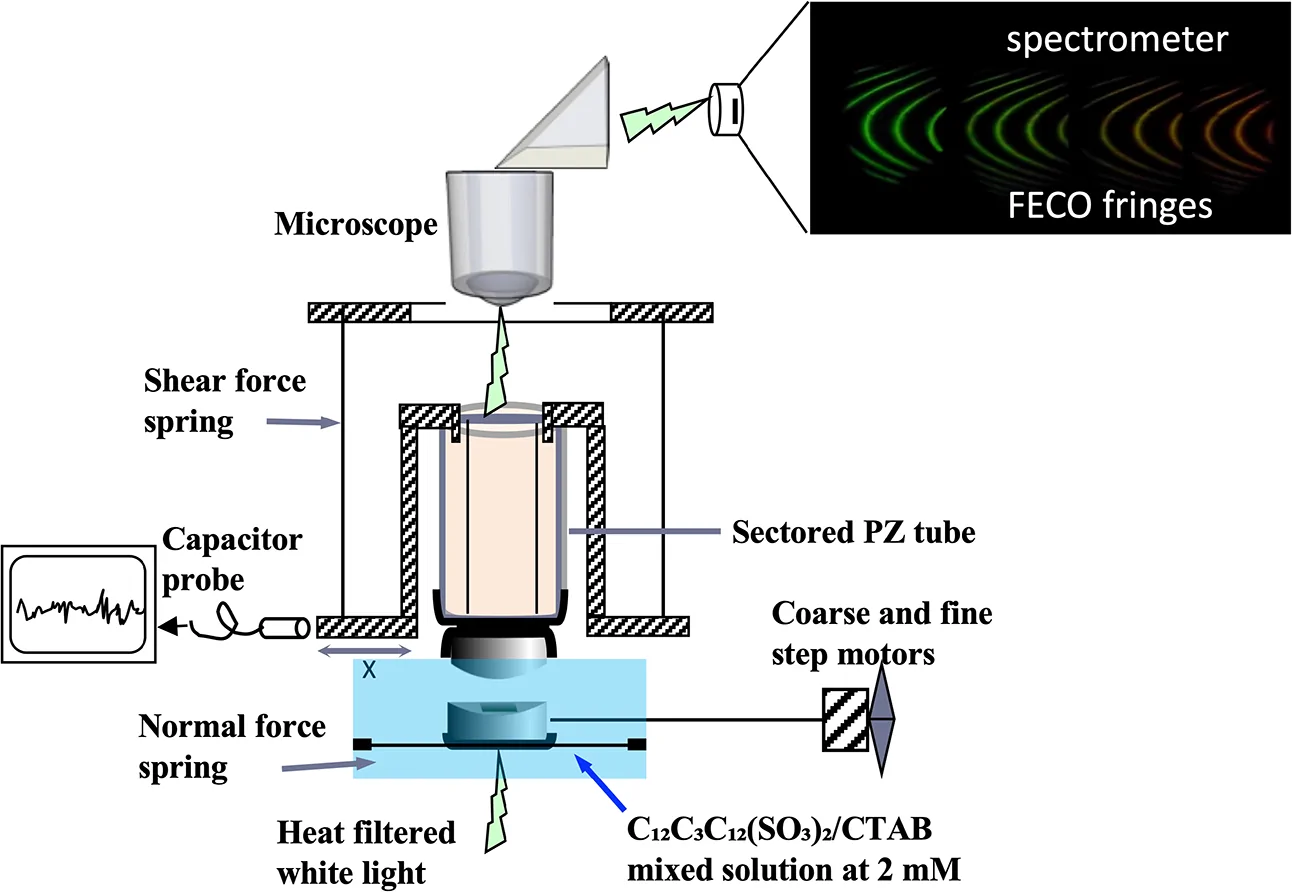
Schematic of the surface force balance setup used to measure normal and shear interactions between two mica surfaces in surfactant dispersions. Multiple-beam interference occurs when white light passes through the semireflective mica layers, producing Fringes of Equal Chromatic Order (FECO; inset, top right). Analysis of these fringes provides the absolute surface separation with a resolution of approximately ±0.2 nm. A segmented piezoelectric tube enables controlled motion of the surfaces in both the normal and lateral directions. The resulting deflections of the normal and shear springs, detected either by variations in an air-gap capacitor probe or by changes in the interference fringes, allow direct determination of the corresponding normal and shear forces. After calibration in both air and pure water, measurements were performed in C_12_C_3_C_12_(SO_3_)_2_/CTAB mixed solutions at varying molar ratios, with the total concentration fixed at 2 mM.

## Results and Discussion

Given that mica is inherently negatively charged in water,^17^ electrostatic repulsion hinders the adsorption of the anionic gemini surfactant C_12_C_3_C_12_(SO_3_)_2_. To accurately characterize the interfacial behavior of its self-assembled aggregates, both AFM and SFB measurements were performed in surfactant dispersions. The hydrodynamic radii of the aggregates at *X*_*g*_ = 0.1, 0.2, and 0.3 were 2.1, 32.9, and 164.3 nm, respectively (Figure 3), consistent with the expected progression from spherical micelles to wormlike micelles and vesicles.^29^

**Figure 3:**
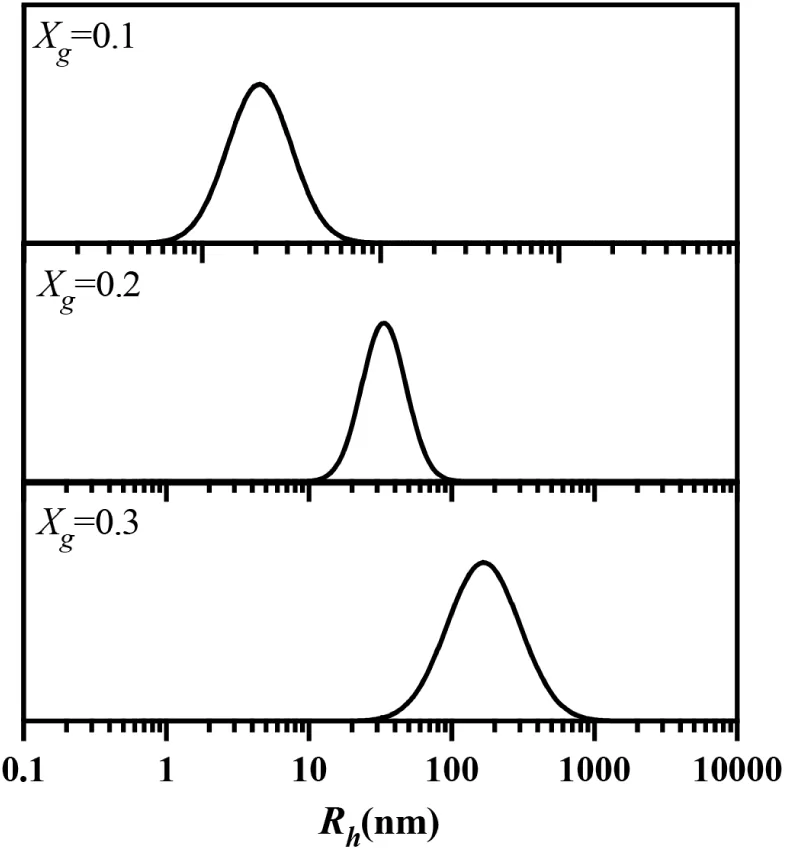
Size distributions of C_12_C_3_C_12_(SO_3_)_2_/CTAB aggregates from DLS at *X_g_* = 0.10, 0.20, and 0.30 at a total surfactant concentration of 2 mM.

The formation of these distinct aggregate morphologies is governed primarily by the anionic/cationic charge ratio.^29^ At low anionic ratio (*X*_*g*_ < 0.16), CTAB is in excess and the mixed aggregates retain a relatively high net positive charge. The resulting electrostatic repulsion between headgroups maintains a high interfacial curvature, favoring the formation of spherical micelles. As *X*_*g*_ increases, the incorporation of the doubly charged anionic gemini surfactant partially neutralizes the cationic headgroups and reduces the effective headgroup area. This promotes micellar growth and leads to wormlike micelles at intermediate *X*_*g*_ (0.16 < *X*_*g*_ < 0.22). When *X*_*g*_ approaches the stoichiometric charge-neutralization point, *X*_*g*_ = 1/3, the net charge density is further reduced, and the surfactant molecules pack more closely, favoring bilayer-type assemblies and vesicle formation.

In the present study, the total surfactant concentration was maintained at 2 mM, a condition for which solution aggregation behavior had been previously established.^29^ As such, independent critical concentrations for each morphology were not determined. Instead, *X*_*g*_ values of 0.1, 0.2, and 0.3 were selected according to the composition-dependent morphology windows described above. Aggregate morphologies had previously been confirmed by direct cryo-TEM, and the DLS measurements obtained in this study further confirmed that the observed aggregate sizes were consistent with these structural assignments.

### Surface Structure Characterization

To characterize the structure of C_12_C_3_C_12_(SO_3_)_2_/CTAB mixtures at the mica–water interface, AFM imaging was performed at different molar ratios. However, high-quality images were obtained only at *X*_*g*_ = 0.2. The lack of stable AFM images at *X*_*g*_ = 0.1 and *X*_*g*_ = 0.3 does not necessarily indicate the absence of surface interaction or adsorption; rather, the small spherical micelles at *X*_*g*_ = 0.1 may be too small or dynamic to resolve, while the large, weakly charged vesicles at *X*_*g*_ = 0.3 may not be sufficiently immobilized on mica during scanning. The interfacial interactions at all three compositions are therefore evaluated primarily from the SFB measurements.

Figure 4A reveals the formation of an extended, laterally homogeneous adsorbed layer of C_12_C_3_C_12_(SO_3_)_2_/CTAB at *X*_*g*_ = 0.2. Higher-magnification imaging (Figure 4B) shows hemimicelles or flattened wormlike micelles aligned with their long axes parallel to one another, with a center-to-center distance (diameter) of about 7.5 nm and only small variation (Figure 4B, inset). CTAB morphology on mica has been investigated extensively at 1.8 mM, 0.8 mM, and 5 mM.^37,38^ The cylindrical structures reported in those studies exhibit a center-to-center distance of 4.2 ± 0.4 nm, approximately twice the length of CTAB, a value substantially smaller than that observed here. This can be interpreted using packing parameter theory:^19^

1 $$P=\frac{v}{a_{0}l_{c}}$$

where ν is the volume of the hydrocarbon core, *a*_0_ is the hydrophilic headgroup area, and *l*_*c*_ represents the length of the hydrocarbon chain. Electrostatic attraction between CTAB and C_12_C_3_C_12_(SO_3_)_2_ reduces the effective headgroup area *a*_0_, thereby increasing the packing parameter and decreasing the spontaneous curvature. This interpretation is consistent with previous studies of cationic–anionic surfactant mixtures, where electrostatic pairing and charge-ratio regulation promote micellar growth and drive the transition from spherical micelles to wormlike micelles and vesicles.^22,25^ The resulting reduction in curvature accounts for the larger micellar cross-sectional diameter (∼7.5 nm) observed by AFM.

**Figure 4:**
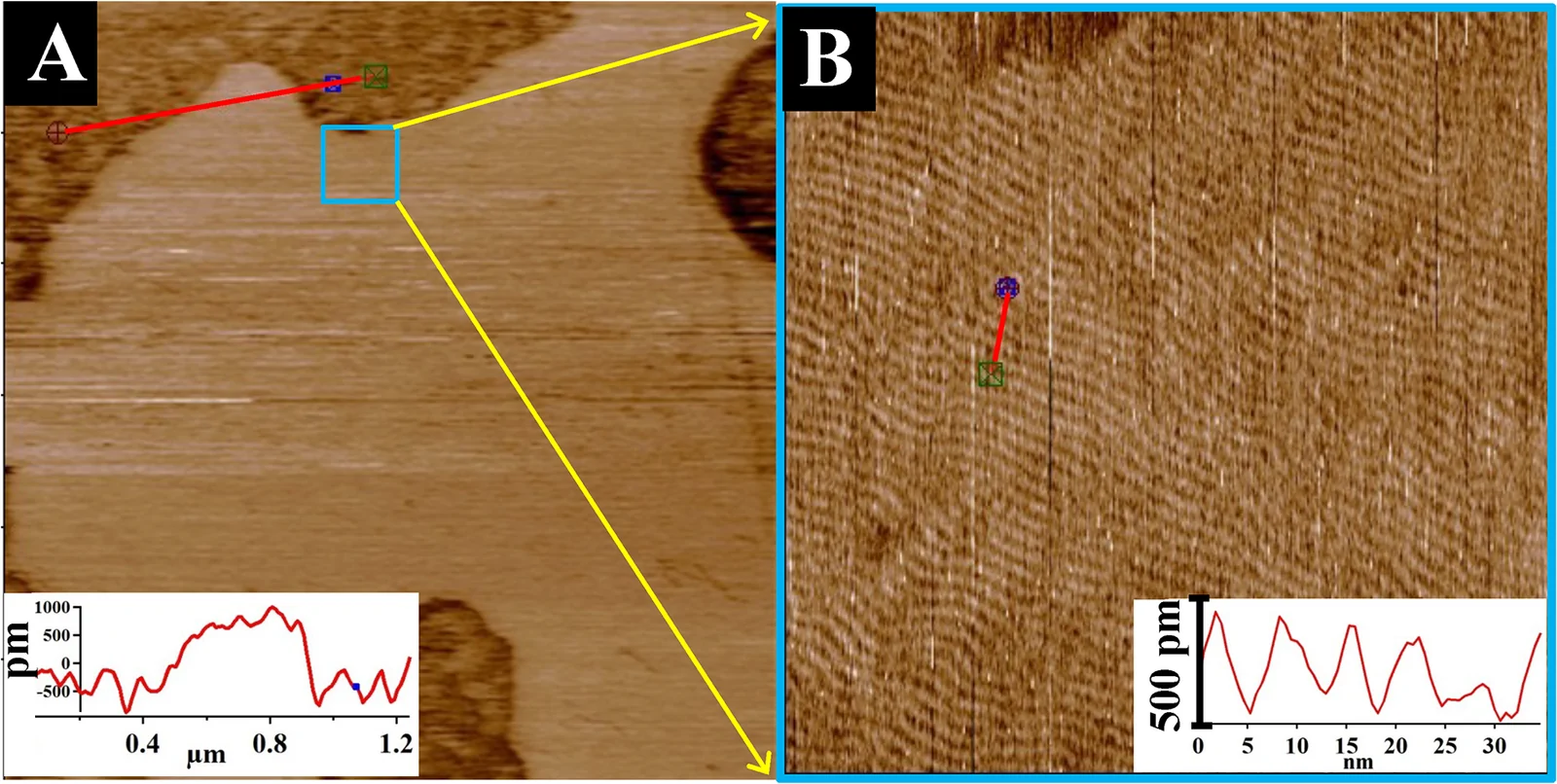
AFM images of mica surfaces following adsorption from C_12_C_3_C_12_(SO_3_)_2_/CTAB mixed solution at *X_g_* = 0.2. (A) Scan area of 5 × 5 μm^2^. (B) Higher-magnification image of the highlighted region in panel A.

This AFM length scale is substantially smaller than the DLS-derived hydrodynamic radius because it represents the local center-to-center spacing or apparent cross-sectional width of flattened surface-bound structures, rather than the overall hydrodynamic size of bulk wormlike micelles. Their mechanical stability under confinement is evaluated from the SFB normal and shear force measurements below.

### Normal Force Interactions between Mica Surfaces across the C_12_C_3_C_12_(SO_3_)_2_/CTAB Solutions at Different Molar Ratios

The normal force properties of C_12_C_3_C_12_(SO_3_)_2_/CTAB solutions at *X*_*g*_ = 0.1, 0.2, and 0.3 confined between mica surfaces were investigated by using the SFB, as shown schematically in Figure 2. Before each experiment, control measurements between bare mica surfaces in conductivity water calibrated the zero separation distance and lateral displacement. Measurements were then conducted on the surfactant dispersions. The resulting normal force profiles, expressed as *F*_*n*_(*D*/*R*) as a function of the surface separation *D*, are shown in Figure 5.

**Figure 5:**
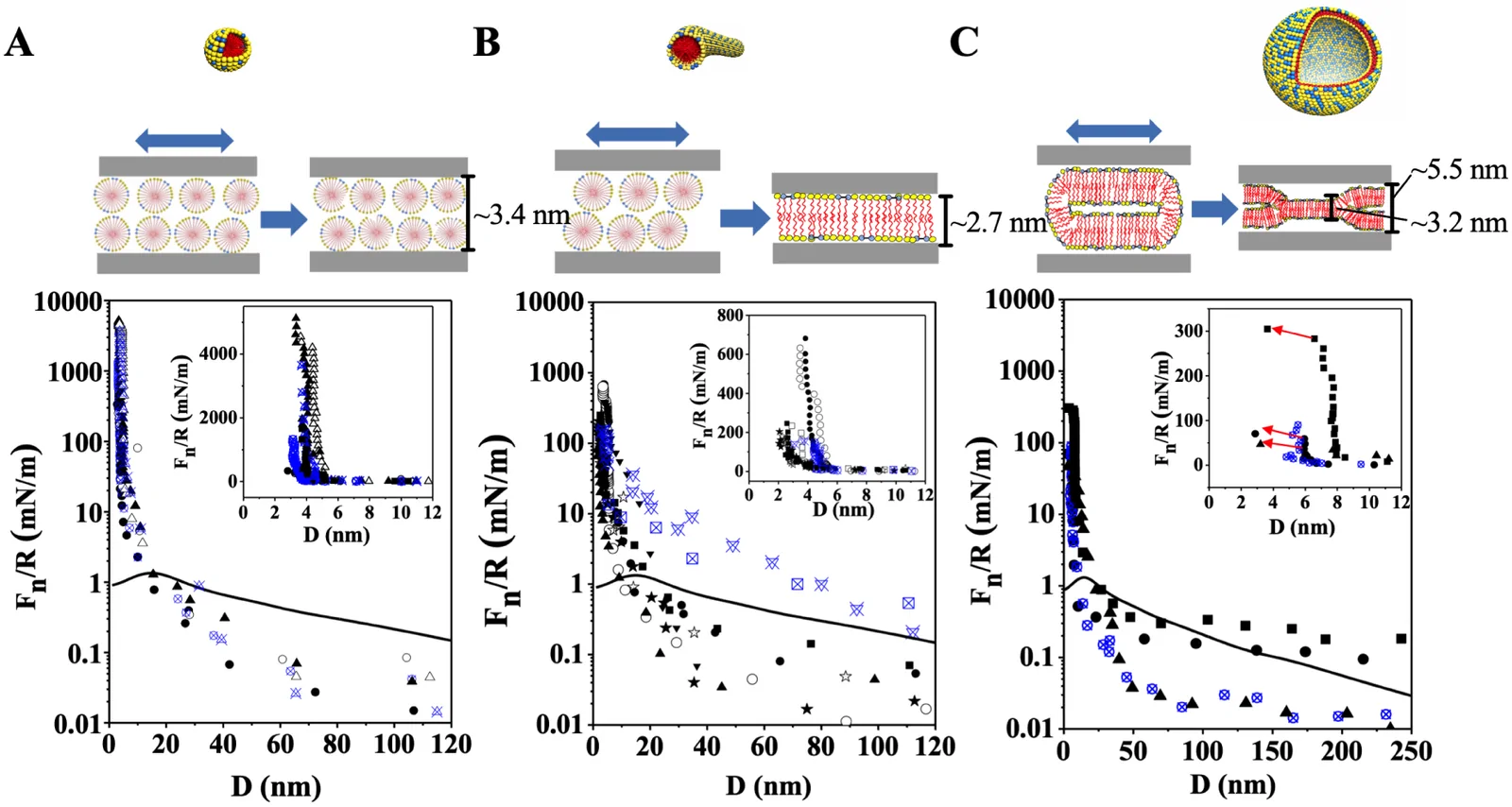
Normal force *F_n_* versus distance *D* profiles of C_12_C_3_C_12_(SO_3_)_2_/CTAB mixed solutions at different molar ratios: (A) *X_g_* = 0.1, (B) *X_g_* = 0.2, and (C) *X_g_* = 0.3. The black curves show the control force profiles measured between bare mica surfaces in pure water. Insets show the corresponding “hard wall” behavior on a linear scale using the same data points as the main panels. Schematic illustrations of the dominant aggregate structures and the molecular arrangement at hard-wall separation at each molar ratio are shown above the plots. For *X_g_* = 0.3, the schematic distinguishes the initial state comprising two opposing compressed vesicle-derived bilayers from the thinner post jump-in hemifusion-like state. Yellow represents the CTAB hydrophilic headgroup, and blue is the headgroup of C_12_C_3_C_12_(SO_3_)_2_. Different symbols represent different contact regions. Filled black symbols represent first approaches, open symbols are receding profiles, and crossed blue symbols indicate second approaches. Red arrows indicate the “jump-in” event. No receding profiles were recorded in panel C because “jump-out” events occurred at all contact points at *X_g_* = 0.3.

The “mechanical stability” in the following discussion refers to the ability of the adsorbed or confined surfactant structures to maintain their force response and limiting separation under repeated compression and shear. It was evaluated from several SFB observables:^16^ the overlap between the first-approach, separation, and second-approach force profiles; the preservation or change of the hard-wall distance; the occurrence of jump-in or jump-out events; and changes in friction during repeated sliding. Thus, stability in this work represents mechanical robustness under confinement, rather than thermodynamic stability of the bulk aggregates.

The far-field decay region of the force curves was analyzed to characterize the electrostatic double-layer repulsion. In this regime, the normal force profiles exhibit smooth exponential decay before short-range steric, hydration, or aggregate-compression forces become significant, and follow the standard form derived from DLVO theory.^39^ Fitting the exponential decay yields the Debye length κ^–1^, which is related to the ionic strength of the medium through

2 $$\kappa^{-1}={\left(\frac{\varepsilon k_{B}T}{8\pi e^{2}c}\right)}^{1/2}$$

where *ε* is the dielectric permittivity of water, *c* is the ion concentration, *e* is the elementary charge, and *k*_B_ and *T* are the Boltzmann constant and absolute temperature, respectively. Importantly, the Debye length reflects the concentration of mobile ions contributing to screening in the confined gap, rather than the total surfactant concentration.

The “hard-wall separation” below represents the limiting surface separation reached at high normal load, where further compression produces a steep increase in normal force. This distance is interpreted as the thickness of the compressed adsorbed or confined surfactant layer remaining between the two mica surfaces. It is therefore distinct from the hydrodynamic radius measured by DLS in bulk solution. The comparison between DLS and SFB thus provides information on confinement-induced deformation and reorganization, rather than a direct size match between free aggregates and confined structures. The pure-water control profiles in Figure 5 are plotted as continuous solid lines, but the data were acquired manually using the same protocol as the surfactant force profiles. The lines connecting the data points are guides to the eye; therefore, the apparent discontinuity before contact should not be interpreted as the measured van der Waals jump-in distance.

#### *X_g_* = 0.1 (Spherical Micelles)

At *X*_*g*_ = 0.1 (Figure 5A), where the C_12_C_3_C_12_(SO_3_)_2_/CTAB mixture forms spherical micelles, the first-approach, separation, and second-approach force profiles largely overlap, demonstrating structural stability under confinement. The onset of long-range repulsion at *D* ≈ 70 nm was substantially shorter than that measured between bare mica surfaces in pure water (approximately 150 nm), used here as a control. This reduced interaction range indicates that the surfactant solution modifies and screens the electrostatic double-layer repulsion between bare negatively charged mica surfaces. In the surfactant dispersion, additional mobile ions, including Br^–^ counterions from CTAB and Na^+^ counterions associated with C_12_C_3_C_12_(SO_3_)_2_, together with ions released or exchanged during aggregate formation and interaction with mica, increase the effective ionic strength and therefore shorten the Debye screening length.^40^ Two distinct regimes were observed. In the far-field region, *F*(*D*)/*R* increased exponentially as the surfaces approached *D* ≈ 10–15 nm, followed by a pronounced increase in repulsion for *D* < 6 nm. At the maximum applied pressure (∼70 atm), the final separation reached *D* = 3.4 ± 0.5 nm. Fitting the far-field region to DLVO theory yielded a Debye length of *κ* ^–1^ = 17.2 ± 0.6 nm, corresponding to an effective salt concentration *c* = 0.31 ± 0.02 mM. Although the total surfactant concentration is 2 mM (1.8 mM CTAB and 0.2 mM C_12_C_3_C_12_(SO_3_)_2_ at *X*_*g*_ = 0.1), the effective mobile ion concentration is substantially lower. The short-range repulsion below ∼6 nm likely arises from steric repulsion from overlapping hydration layers and compression of adsorbed surfactant structures.

#### *X_g_* = 0.2 (Wormlike Micelles)

At *X*_*g*_ = 0.2 (Figure 5B), where wormlike micelles are formed, the range of electrostatic repulsion decreases to *D* ≈ 40 nm and the exponential far-field regime reduces to *D* ≈ 5 nm. The maximum applied pressure decreases to ∼37 atm and the hard-wall distance is *D* = 2.7 ± 0.8 nm (or 3.9 ± 0.3, depending on the contact region), consistent with the thickness of the bilayer between mica surfaces. Fitting the first-approach curves gives *κ^–^* ^1^ = 5.4 ± 0.3 nm, corresponding to an effective ionic strength of ∼3.2 mM. The shorter Debye length indicates a higher concentration of mobile ions in the gap than at *X*_*g*_ = 0.1. The second-approach curve (blue symbols) shifts outward and exhibits stronger long-range repulsion than the first approach. This suggests that the first compression/shear cycle modifies the surface-bound wormlike micellar layer through flattening, redistribution, structural rearrangement, and/or partial damage. Similar changes in repeated SFB approach profiles have been associated with deformation or reorganization of soft surfactant/lipid boundary layers under confinement.^41^

At constant total surfactant concentration (2 mM), the longer Debye length at *X*_*g*_ = 0.1 reflects the combined effects of charge neutralization, counterion condensation, and charge compensation within the gap by net positively charged aggregates. At *X*_*g*_ = 0.1, CTAB is in substantial excess and preferentially adsorbs onto the negatively charged mica surfaces, leading to charge compensation and, potentially, overcompensation.^16^ Surface overcharging can promote Br^–^ association in the Stern layer adjacent to the mica, effectively trapping counterions close to the surface.^42,43^ In addition, compact spherical micelles may exhibit stronger counterion condensation and more limited monomer exchange than do wormlike micelles. Consequently, the spherical micelle solution exhibits a lower concentration of freely mobile ions in the gap and a longer Debye screening length.

At *X*_*g*_ = 0.2, the composition is closer to the 2:1 stoichiometric neutralization condition. A larger fraction of CTAB participates in cation–anion pairing, increasing the pool of small mobile ions released from individual charged headgroups. In parallel, the extended wormlike micelles undergo dynamic scission–recombination,^44,45^ which can reduce counterion condensation relative to compact spheres and enhance ion exchange with the surrounding solution. Because more CTAB is consumed in bulk neutralization, surface overcharging and Stern-layer counterion association are also expected to be reduced relative to *X*_g_ = 0.1. Together, these effects account for a higher mobile-ion concentration in the gap and a shorter *κ* ^–1^.

#### *X_g_* = 0.3 (Vesicles)

At *X*_*g*_ = 0.3, corresponding to vesicle-dominated solutions, the force profiles exhibit a pronounced long-range steric contribution extending from approximately 50 to 250 nm (Figure 5C). This repulsion arises from the loosely adsorbed vesicle layers and from the confinement and deformation of vesicles trapped between the mica surfaces. Although the hydrodynamic radius of the vesicles in bulk solution is much larger than that of the spherical or wormlike micelles, the limiting separation measured by SFB does not correspond to the diameter of intact vesicles. Under confinement, vesicles are expected to deform, flatten, expel internal water, and partially rupture or reorganize into bilayer-like structures. Thus, the hard-wall separation of 5.5 ± 0.9 nm reflects the thickness of two opposing compressed vesicle-derived bilayers, rather than the size of the original bulk vesicles. Since steric and electrostatic interactions overlap, the normal force profiles cannot be described quantitatively by classical DLVO theory, and no Debye length was fitted for this composition.

At a critical applied pressure, a jump-in event occurs (indicated by red arrows), reducing the separation to 3.2 ± 0.4 nm and producing rigid coupling. We attribute this transition to a hemifusion-like rearrangement between the two opposing, flattened vesicle-derived bilayers. In this intermediate fusion state, the outer leaflets of the apposed bilayers merge to form a continuous film, whereas the inner leaflets remain distinct.^46^ Such a rearrangement would convert the initially thicker, two-bilayer structure into one fused bilayer. The subsequent adhesive contact and jump-out upon separation are consistent with this irreversible structural transition. Subsequent compression cycles no longer show the original long-range repulsion, indicating that the vesicles responsible for this interaction were irreversibly deformed, ruptured, expelled, or reorganized during the first compression cycle.

Overall, the normal force profiles reveal distinct confinement responses for the three morphologies. At *X*_*g*_ = 0.1, the first approach, separation, and second approach profiles of the spherical micelle layers largely overlap, indicating that the adsorbed layer formed from spherical micelles is mechanically stable. The wormlike layer at *X*_*g*_ = 0.2 undergoes a compression-induced rearrangement. At *X*_*g*_ = 0.3, large vesicles provide long-range steric repulsion on the first approach but collapse irreversibly into a thinner adhesive-bilayer-derived structure under load.

### Lubrication Properties of C_12_C_3_C_12_(SO_3_)_2_/CTAB Mixtures at Different Molar Ratios on Mica Surfaces

Shear force (*F*_*s*_) measurements were performed by applying lateral displacement to the upper SFB surface while maintaining the lower surface fixed. Typical shear traces are presented in panel A of Figures 6–8. The triangular waveforms represent the applied lateral displacement, Δ*x*_0_(*t*), as a function of time (*t*), and the lower traces show the measured shear force, *F*_*s*_(*t*). Dynamic sliding friction is indicated by the plateaus in *F*_*s*_. Under applied normal pressures, where the magnitude of *F*_*s*_ was comparable to the instrumental noise and distinct plateaus were not resolved, dynamic sliding friction was quantified by fast Fourier transform (FFT) analysis at the drive frequency.

**Figure 6:**
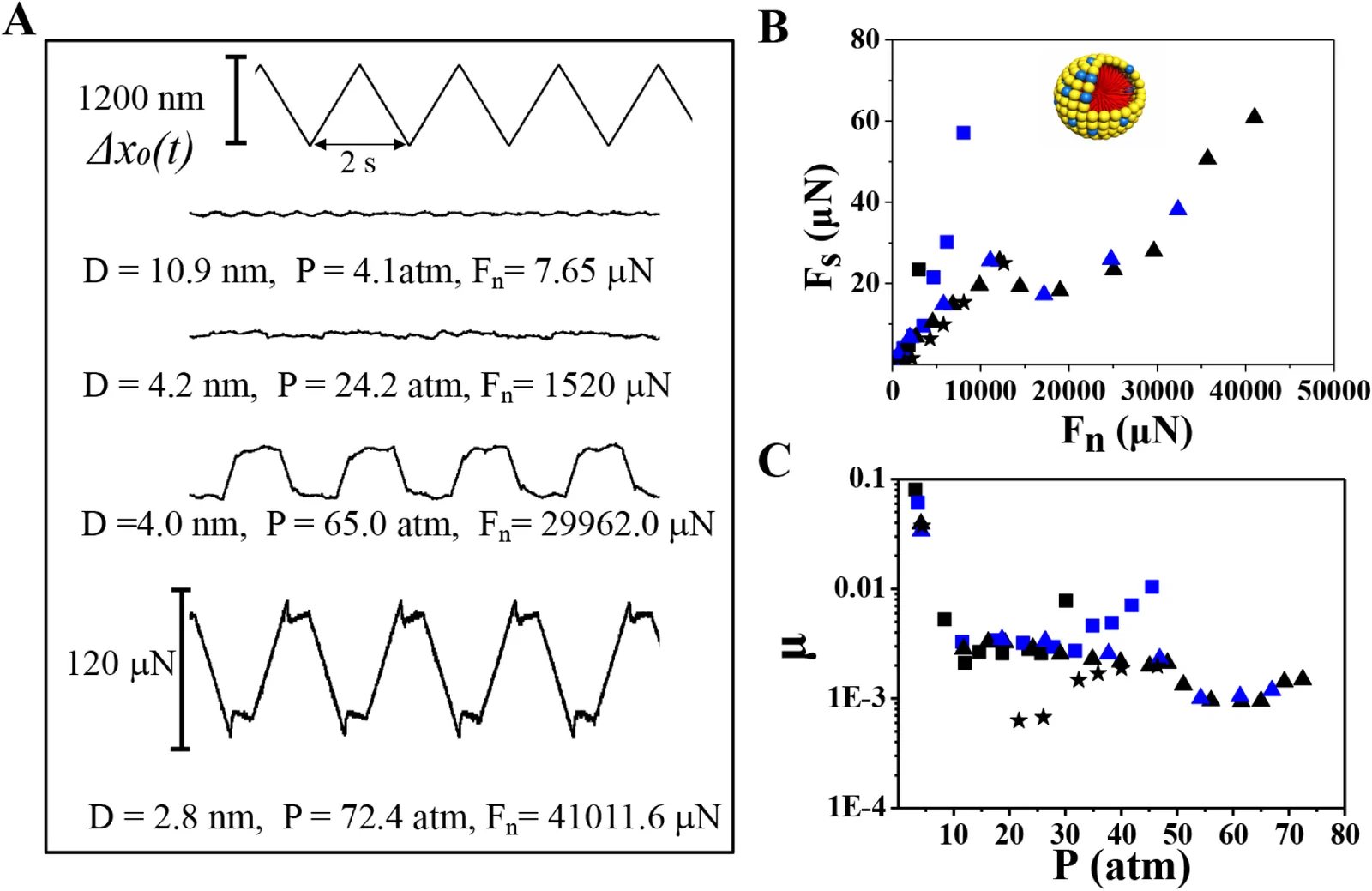
Shear force profiles of C_12_C_3_C_12_(SO_3_)_2_/CTAB mixed solution at *X_g_* = 0.1. (A) Shear force *F_s_* versus time traces. The upper trace is the applied lateral displacement Δ*x*_0_(*t*) of the upper mica surface, driven by the piezoelectric tube, relative to the lower mica surface. (B) Shear force *F_s_* as a function of applied load *F_n_*. (C) Corresponding friction coefficient μ versus pressure *P* for the data in panel (B). Different symbols represent different contact regions. Black symbols represent first approaches, and blue symbols represent second approaches.

The stick–slip character of the measured shear traces should be interpreted as the mechanical response of the confined surfactant layers under load. It is governed by the coupling between the opposing surfactant-covered mica surfaces, the hydration and mobility of the charged headgroups, the load-bearing ability of the confined layer, and whether the layer remains intact or undergoes rearrangement or collapse during compression and sliding. Therefore, a more discrete aggregate morphology does not necessarily lead to stronger stick–slip; instead, highly hydrated and weakly coupled interfacial layers can slide smoothly even when they originate from larger or more discrete aggregates.

At each surface separation, the mean contact pressure *P* was calculated as *P = F*_*n*_/*A*, where *A* = π*a* ^2^ is the contact area. When FECO fringes showed clear flattening, *a* was determined as the mean radius of the circular or elliptical contact. At lower loads, where no clear flattening was observed, the Hertz relation,^47^ *a* ^3^ = *F*_*n*_ *R*/*K*, was used with *K* = (5 ± 2) × 10^9^ N/m^2^ for the effective elastic modulus of the mica/glue combination.^15^ Because *A* increases with *F*_*n*_, pressure does not scale linearly with the normal force. In the Hertzian limit, *P = F*_*n*_/π*a* ^2^ scales approximately as *F*_*n*_^1/3^. The friction coefficient at a given pressure *P* was calculated as μ = *F*_*s*_/*F*_*n*_. Panel B and C of Figures 6–8 show *F*_*s*_ versus *F*_*n*_ and *μ* versus *P*, respectively.

#### *X_g_* = 0.1 (Spherical Micelles)

In the spherical micelle solution (Figure 6), the friction coefficient decreases with increasing normal load and reaches μ ≈ 0.001–0.003, remaining in this range up to pressures of 50–72 atm. Below ∼25 atm, the shear force is very small (<5 μN, Figure 6A). Above this threshold, the shear force increases gradually with further loading. At a critical pressure, rigid coupling occurs at a separation *D* = 3.4 ± 0.5 nm. The ultralow friction is attributed to lubrication.^48^ The quaternary ammonium and sulfonate headgroups are strongly hydrated and form robust hydration shells that resist squeeze-out under load. Previous studies of C_12_C_3_C_12_(SO_3_)_2_-containing aggregates suggest that water-mediated interactions contribute to their self-assembly, while studies of sulfonate-containing amphiphiles have shown that sulfonate groups support structured interfacial hydration shells.^34,48–50^ CTAB-rich layers adsorb strongly onto negatively charged mica, providing electrostatic stabilization and structural integrity. Rapid exchange of water within the hydration layers preserves interfacial fluidity and enables low-dissipation shear.^17^ The overlap of the force profiles indicates that the spherical micelle-derived layer largely recovers after unloading, preserving low friction over repeated cycles.

#### *X_g_* = 0.2 (Wormlike Micelles)

At *X*_*g*_ = 0.2 (Figure 7), the friction coefficient remains low (μ ≈ 0.002–0.003), but the maximum sustainable pressure decreases to ∼30–40 atm. In most contact regions, the hard-wall separation decreases from 3.9 ± 0.3 nm to 2.7 ± 0.8 nm at the critical pressure, indicating compression and partial collapse of the confined wormlike aggregates. The reduced load-bearing capacity is consistent with a lower effective surface charge density caused by partial charge neutralization. Weaker electrostatic repulsion and structural instability of cylindrical aggregates under high confinement increase their susceptibility to shear-induced damage. Consistent with this interpretation, the second-approach normal-force profiles (Figure 5B, blue symbols) show enhanced long-range repulsion, accompanied by a significantly higher friction coefficient (Figure 7B and C), indicating damage, reorganization, or partial depletion of the surface-bound aggregates.^1^

**Figure 7:**
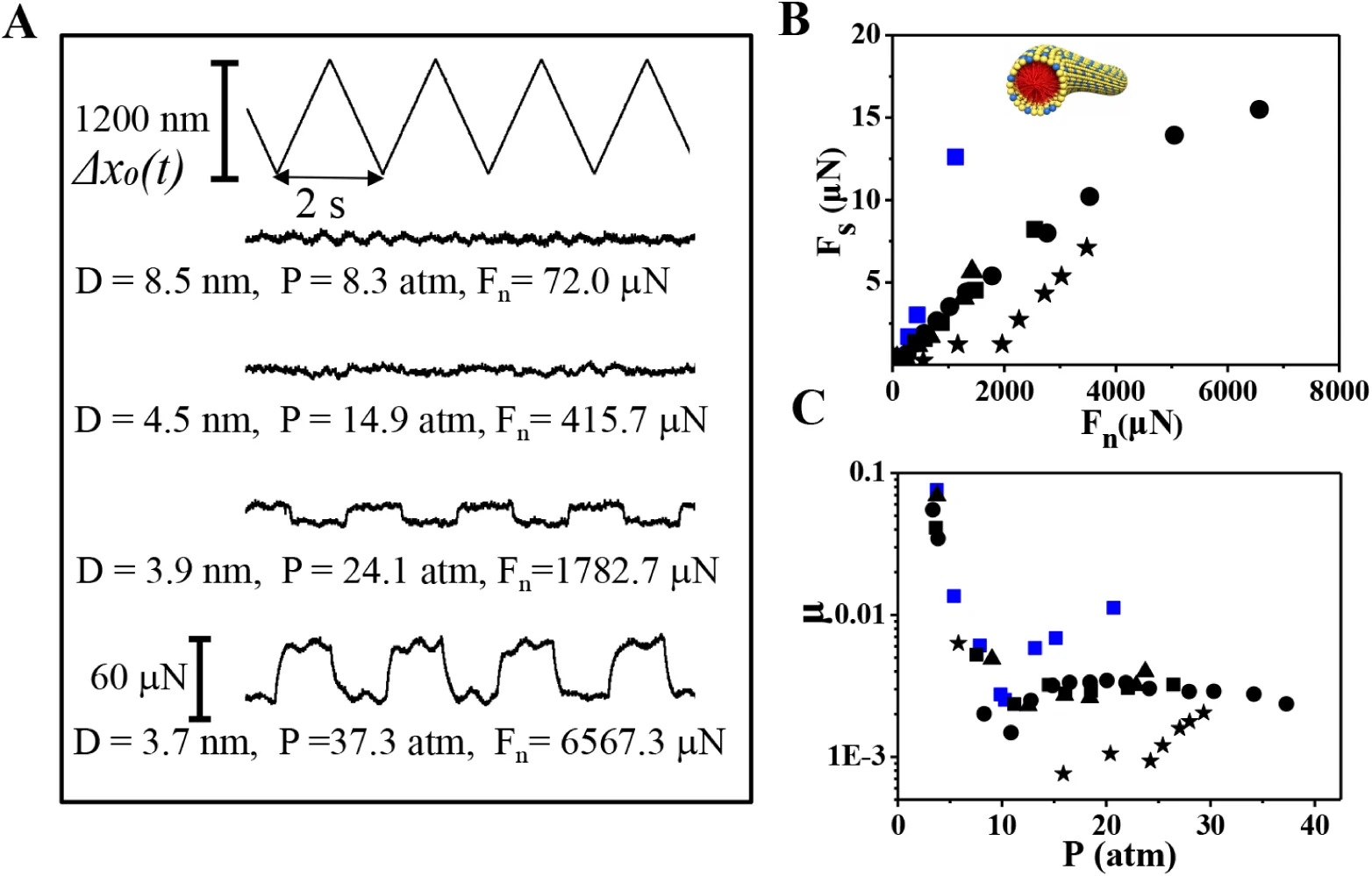
Shear force profiles of C_12_C_3_C_12_(SO_3_)_2_/CTAB mixed solution at *X_g_* = 0.2. (A) Shear force *F_s_* versus time traces. The upper trace is the applied lateral displacement Δ*x*_0_(*t*). (B) Shear force *F_s_* as a function of applied load *F_n_*. (C) Corresponding friction coefficient μ versus pressure *P* for the data in panel (B). Different symbols represent different contact regions. Filled black symbols represent first approaches, and blue symbols represent second approaches.

#### *X_g_* = 0.3 (Vesicles)

At *X*_*g*_ = 0.3 (Figure 8), the friction coefficient reaches μ ≈ 10^–4^, the lowest among the three C_12_C_3_C_12_(SO_3_)_2_/CTAB ratios. Vesicles initially act as soft, deformable load-bearing units at large separations. On compression to *D* = 5.5 ± 0.9 nm, they form flattened bilayers on the mica surfaces, providing highly hydrated interfaces. Shear forces are extremely small (0.25–1 μN, Figure 8A), close to the systematic background signal arising from coupling to the piezocrystal wires.^15^ The lowest friction near the charge neutrality suggests that reduced net surface charge weakens electrostatic pinning between the surfactant layer and mica, while ammonium and sulfonate headgroups retain strong hydration shells. This reduced interfacial coupling, together with symmetric hydration layers, minimizes dissipative interactions and yields an order-of-magnitude lower friction coefficient than at other compositions. However, mechanical stability is limited at *X*_*g*_ = 0.3. At 15–30 atm, the two opposing flattened vesicle-derived bilayers undergo the hemifusion-like rearrangement described above, reducing the separation from 5.5 ± 0.9 nm to 3.2 ± 0.4 nm and leading to surface-layer collapse, adhesive contact, and rigid coupling. Although the postjump-in separation at *X*_*g*_ = 0.3 is similar to the limiting separation at *X*_*g*_ = 0.2, the two states should not be regarded as structurally or tribologically equivalent. The ultralow-friction data *X*_*g*_ = 0.3 predominantly correspond to the prejump-in flattened vesicle-derived bilayers, whereas the jump-in produces adhesive contact and rigid coupling, preventing measurement of a comparable stable high-pressure sliding branch.

**Figure 8:**
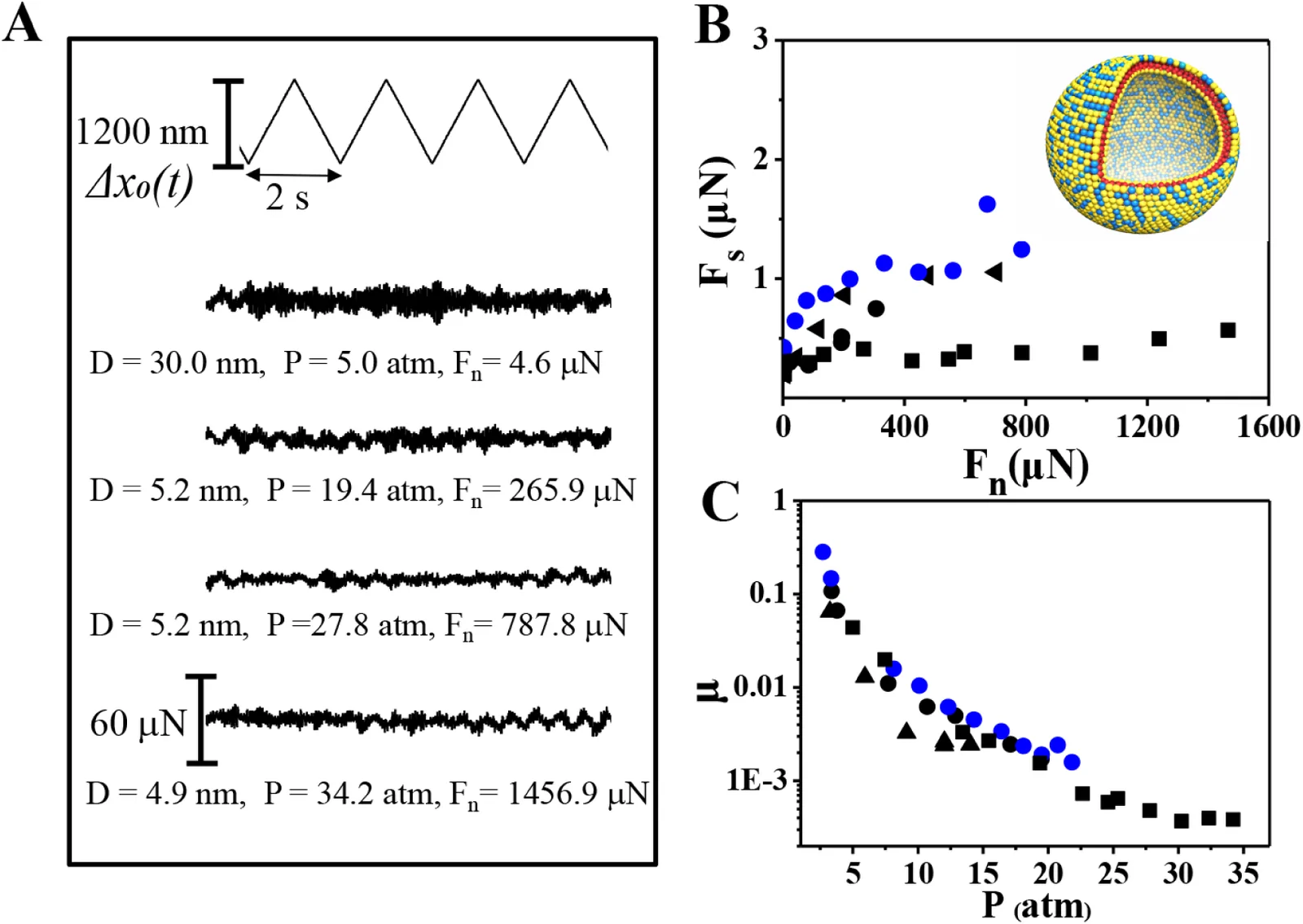
Shear force profiles of C_12_C_3_C_12_(SO_3_)_2_/CTAB mixed solution at *X_g_* = 0.3. (A) Shear force *F_s_* versus time traces. The upper trace is the applied lateral displacement Δ*x*_0_(*t*). (B) Shear force *F_s_* as a function of applied load *F_n_*. (C) Corresponding friction coefficient μ versus pressure *P* for the data in panel B. Different symbols represent different contact regions. Filled black symbols represent first approaches, and blue symbols represent second approaches.

The apparent morphology-dependent friction reflects a balance among hydration lubrication, charge neutralization, and mechanical stability. From *X*_*g*_ = 0.1 to *X*_*g*_ = 0.3, the aggregates become progressively closer to charge neutralization. At *X*_*g*_ = 0.1, the CTAB-rich spherical micelles provide strong adsorption onto negatively charged mica and form robust interfacial layers, explaining their superior load-bearing stability. However, the stronger electrostatic coupling also gives slightly higher friction than the vesicle system. At *X*_*g*_ = 0.2, partial charge neutralization promotes wormlike micelle formation but the confined layers are more susceptible to compression- and shear-induced rearrangements, leading to reduced mechanical robustness. At *X*_*g*_ = 0.3, the vesicle system is closest to charge neutrality among the three compositions, which reduces electrostatic pinning at the interface and gives the lowest friction coefficient. Nevertheless, the same reduction in charge and the soft vesicular structure also lower the mechanical stability, leading to collapse under moderate pressure. Thus, spherical micelles provide the best load-bearing lubrication, whereas vesicles provide the lowest friction and poorer durability.

Mixed surfactant systems provide an effective strategy for tuning aggregate morphology without increasing total surfactant concentration. This approach avoids common issues associated with high surfactant concentrations, including precipitation, excessive viscosity, and increased formulation cost. In the absence of additives, a CTAB solution undergoes a sphere-to-rod transition only above approximately 250 mM,^51^ whereas the gemini surfactant C_12_C_3_C_12_(SO_3_)_2_ forms spherical micelles below 2 mM.^32^ Remarkably, the C_12_C_3_C_12_(SO_3_)_2_/CTAB mixtures form a rich range of aggregate structures at a total concentration of only 2 mM, 1 to 2 orders of magnitude below the concentrations required for morphological transitions in the individual components. Moreover, the mixed system exhibits ultralow friction coefficients comparable to those achieved using significantly higher concentrations (∼13 mM) of single-chain surfactants such as CTAC.^16^ These results demonstrate that the catanionic surfactant system is a molecularly efficient platform for aqueous lubrication. The molar ratio controls stoichiometric charge neutralization, and thereby both aggregate morphology and tribological performance. Accordingly, the formulation can be tailored either for high-load operation or for extremely low shear force. More broadly, the results show that the tribological response of soft interfacial layers emerges from coupled bulk self-assembly, confinement-induced structural rearrangement, and interfacial hydration dynamics.

## Conclusions

In this study, we systematically investigated the relationship between aggregate structure and frictional performance in C_12_C_3_C_12_(SO_3_)_2_/CTAB surfactant mixtures. At a fixed total surfactant concentration of 2 mM, varying the molar fraction of C_12_C_3_C_12_(SO_3_)_2_ enabled controlled transitions from spherical micelles to wormlike micelles and vesicles. All three compositions exhibited ultralow friction coefficients (10^–3^–10^–4^) through hydration lubrication, but their load-bearing capacities differed significantly. Spherical micelles provided the highest mechanical stability and robust frictional behavior under high load. Wormlike micelles displayed reduced mechanical robustness and underwent confinement-induced reorganization, whereas vesicles yielded the lowest shear force but collapsed at moderate pressure.

These results demonstrate that aggregate morphology and charge neutralization, rather than surfactant molecular structure alone, play important roles in balancing hydration lubrication against mechanical durability. Catanionic gemini surfactant formulations enable this structural tuning at a low total concentration without added salt or polymeric additives. Because only a small fraction of the gemini surfactant is required to induce morphological transitions, this strategy reduces the reliance on high concentrations of specialty surfactants while retaining excellent lubrication efficiency. Overall, this work establishes mixed surfactant self-assembly as a practical and cost-effective route toward tunable water-based lubricants with controllable friction and load-bearing performance.
